# ﻿Two new species of the subgenus *Indoribates* (*Haplozetes*) (Acari, Oribatida, Haplozetidae) from Tajikistan

**DOI:** 10.3897/zookeys.1229.130103

**Published:** 2025-02-27

**Authors:** Cheng-Lin Liu, Abdulaziz Dalatov, Abdusattor Saidov, Jun Chen

**Affiliations:** 1 Key Laboratory of Zoological Systematics and Evolution, Institute of Zoology, Chinese Academy of Sciences, Beijing, 100101, China Institute of Zoology, Chinese Academy of Sciences Beijing China; 2 College of Life Sciences, University of Chinese Academy of Sciences, Beijing, 100049, China University of Chinese Academy of Sciences Beijing China; 3 E.N. Pavlovsky Institute of Zoology and Parasitology, National Academy of Sciences of Tajikistan, Dushanbe, 73402, Tajikistan E.N. Pavlovsky Institute of Zoology and Parasitology, National Academy of Sciences of Tajikistan Dushanbe Tajikistan

**Keywords:** Morphology, new species, new record, oribatid mites, taxonomy

## Abstract

Two new species of oribatid mites of the subgenus Indoribates (Haplozetes) (Oribatida, Haplozetidae) are described based on adult specimens from Tajikistan. Indoribates (Haplozetes) tajikistanensis**sp. nov.** differs from I. (H.) vindobonensis by the position of the adanal lyrifissure and the shape of the saccule *S2*. Indoribates (Haplozetes) asetosus**sp. nov.** differs from I. (H.) fusifer (Berlese, 1908) by the absence of φ on tibia IV and the shape of epimeral setae and adanal setae *ad*_1-2_; from I. (H.) triungulatus (Beck, 1964) it differs by the length of the interlamellar seta, adanal setae *ad*_1-2_ and tutorium, and the shape of notogastral seta. The genus *Indoribates* is recorded in Tajikistan for the first time.

## ﻿Introduction

The genus *Indoribates* was proposed by Jacot (1929) with *Protoribatespunctulatus* Sellnick, 1925 as the type species. The main generic characters of this genus were summarized by Jacot (1929), Hammer (1979: as for *Sundazetes* Hammer, 1979), Mahunka (1977: as for *Nixozetes* Mahunka, 1977; 1997: as for *Bolkiah* Mahunka, 1997), Balogh and Balogh (1992) and Subías and Shtanchaeva (2023). At present, Subías’s world catalogue of oribatid mite records that the genus *Indoribates* includes five subgenera: the nominate subgenus; *Bihaplozetes* Subías, 2020; *Haplozetes* Willmann, 1935; *Mancoribates* Hammer, 1961, and *Neoindoribates* Subías, 2020 (Subías 2022, 2024 updated). Some authors support the independence of some genera (e.g. *Indoribates*, *Haplozetes*, *Mancoribates*; see Weigmann 2006; Ermilov and Behan-Pelletier 2024; Ermilov et al. 2024; Norton and Ermilov 2024), but we follow the world catalogue of Subías. Xu et al. (2024) proposed that Bihaplozetes is a junior synonym of the subgenus Indoribates to avoid identification confusion. The subgenus Indoribates (Haplozetes) includes 16 species and one subspecies, which have a nearly cosmopolitan distribution (except Antarctica) (Subías 2022, 2024 updated).

The first data on oribatid mite fauna (Acari, Oribatida) of Tajikistan were presented by Bulanova-Zachvatkina (1960a, 1960b). Subsequent studies of oribatids in Tajikistan during the next 64 years were fragmentary but include the works of Ilyasov (1963, 1970), Krivolutsky (1966, 1971), Khristov (1973), Sitnikova (1973), Ghilarov and Krivolutsky (1975), Gordeeva (1980), Karppinen et al. (1986), Ryabinin (1986), Shtanchaeva (2001), Penttinen and Gordeeva (2009), Niedbała (2012), Sidorchuk and Norton (2016), and Wei and Chen (2017).

During taxonomic survey of oribatid mites (Acari, Oribatida) collected from litter and upper soil stratum in Tajikistan, we found two new species of the subgenus Indoribates (Haplozetes), I. (H.) tajikistanensis sp. nov. and I. (H.) asetosus sp. nov. The main goal of this paper is to describe and illustrate these new species. Before the present study, the genus *Indoribates* had not been reported from Tajikistan.

## ﻿Methods

Specimens were temporarily mounted in lactic acid on cavity slides for measurement and illustration. All specimens were examined under a differential interference contrast microscope (Leica DM 2500). Drawings were made with a camera lucida using a Leica transmission light microscope (Leica DM 2500). All specimens were stored in test tubes filled with 75% ethanol solution and deposited in the Institute of Zoology, Chinese Academy of Sciences, Beijing (**IZAS**).

Body length was measured in lateral (temporary cavity slides) or dorsal (permanent slides) view, from the tip of the rostrum to the posterior edge of the notogaster. Notogastral width refers to the maximum width of the notogaster in dorsal view (behind pteromorphs). Lengths of body setae were measured in lateral aspect. All body measurements are presented in micrometers (μm). Formulas for leg setation are given in parentheses according to the sequence trochanter-femur-genu-tibia-tarsus (famulus included). Formulas for leg solenidia are given in square brackets according to the sequence genu-tibia-tarsus.

Morphological terminology used in this paper mostly follows that of papers on *Haplozetes* (Grandjean 1936), and Norton (1977) for leg setal nomenclature. See Norton and Behan-Pelletier (2009) for an overview.

The following abbreviations are used: *lam* = lamella; *slam* = sublamella; *tu* = tutorium; *ro*, *le*, *in*, *bs*, *ex* = rostral, lamellar, interlamellar, bothridial and exobothridial setae, respectively; *Al* = sublamellar porose area; *D* = dorsophragma; *P* = pleurophragma; *c*, *la*, *lm*, *lp*, *h*, *p* = notogastral setae; *Sa*, *S1*, *S2*, *S3* = notogastral saccules; *ia*, *im*, *ip*, *ih*, *ips* = notogastral lyrifissures; *Ad* = sejugal porose area; *gla* = opisthonotal gland opening; *a*, *m*, *h* = subcapitular setae; *or* = adoral seta; *sup*, *inf*, *v*, *l*, *d*, *cm*, *acm*, *ul*, *su*, *vt*, *lt* = palp setae; ω = palp solenidion; *cha*, *chb* = cheliceral setae; *Tg* = Trägårdh’s organ; *Pd I*, *Pd II* = pedotecta I, II, respectively; *1a*, *1b*, *1c*, *2a*, *3a*, *3b*, *3c*, *4a*, *4b*, *4c* = epimeral setae; *dis* = discidium; *cp* = circumpedal carina; *g*, *ag*, *an*, *ad* = genital, aggenital, anal and adanal setae, respectively; *iad* = adanal lyrifissure; *Amar* = marginal porose area; *Tr*, *Fe*, *Ge*, *Ti*, *Ta* = trochanter, femur, genu, tibia, and tarsus, respectively; ω, σ, φ = leg solenidia; ε = leg famulus; *v*, *ev*, *bv*, *l*, *d*, *ft*, *tc*, *it*, *p*, *u*, *a*, *s*, *pv*, *pl* = leg setae; *pa* = porose area.

## ﻿Descriptions

### ﻿ Indoribates (Haplozetes) tajikistanensis sp. nov.

Taxon classificationAnimaliaOribatidaHaplozetidae

428E9F2A-73BA-5D26-A2AB-393B35AF0AAF

https://zoobank.org/49B9B555-B32A-4EFA-90F8-9B4F24CB655B

**Figure 1.**
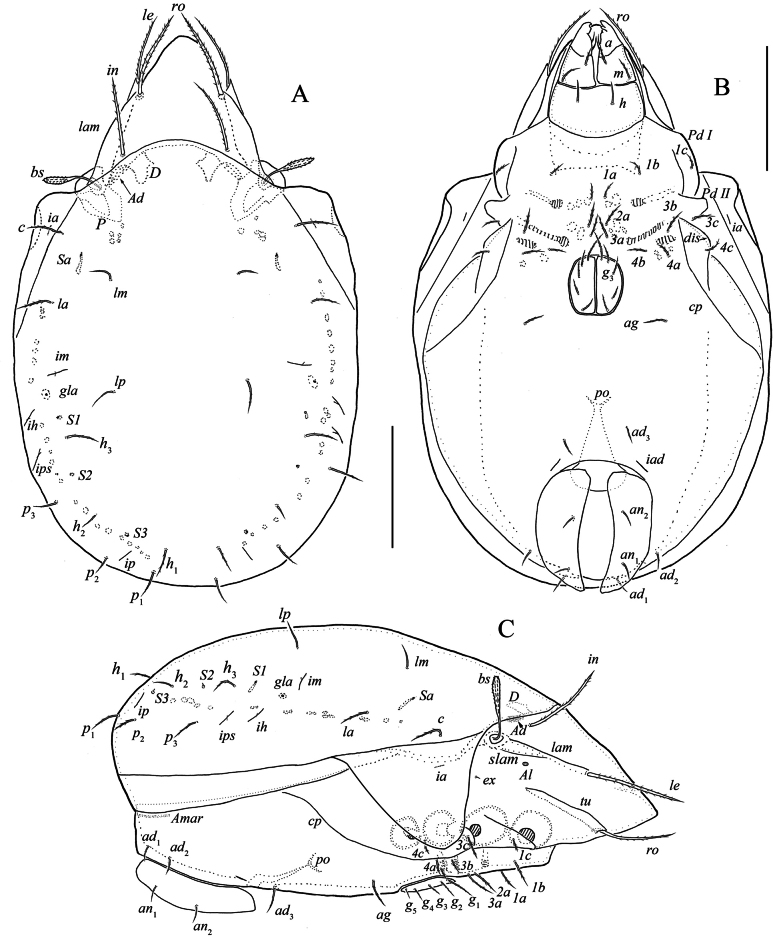
Indoribates (Haplozetes) tajikistanensis sp. nov. **A** dorsal view (legs not shown) **B** ventral view (gnathosoma and legs not shown) **C** lateral view (gnathosoma and legs not shown). Scale bar: 100 μm.

**Figure 2.**
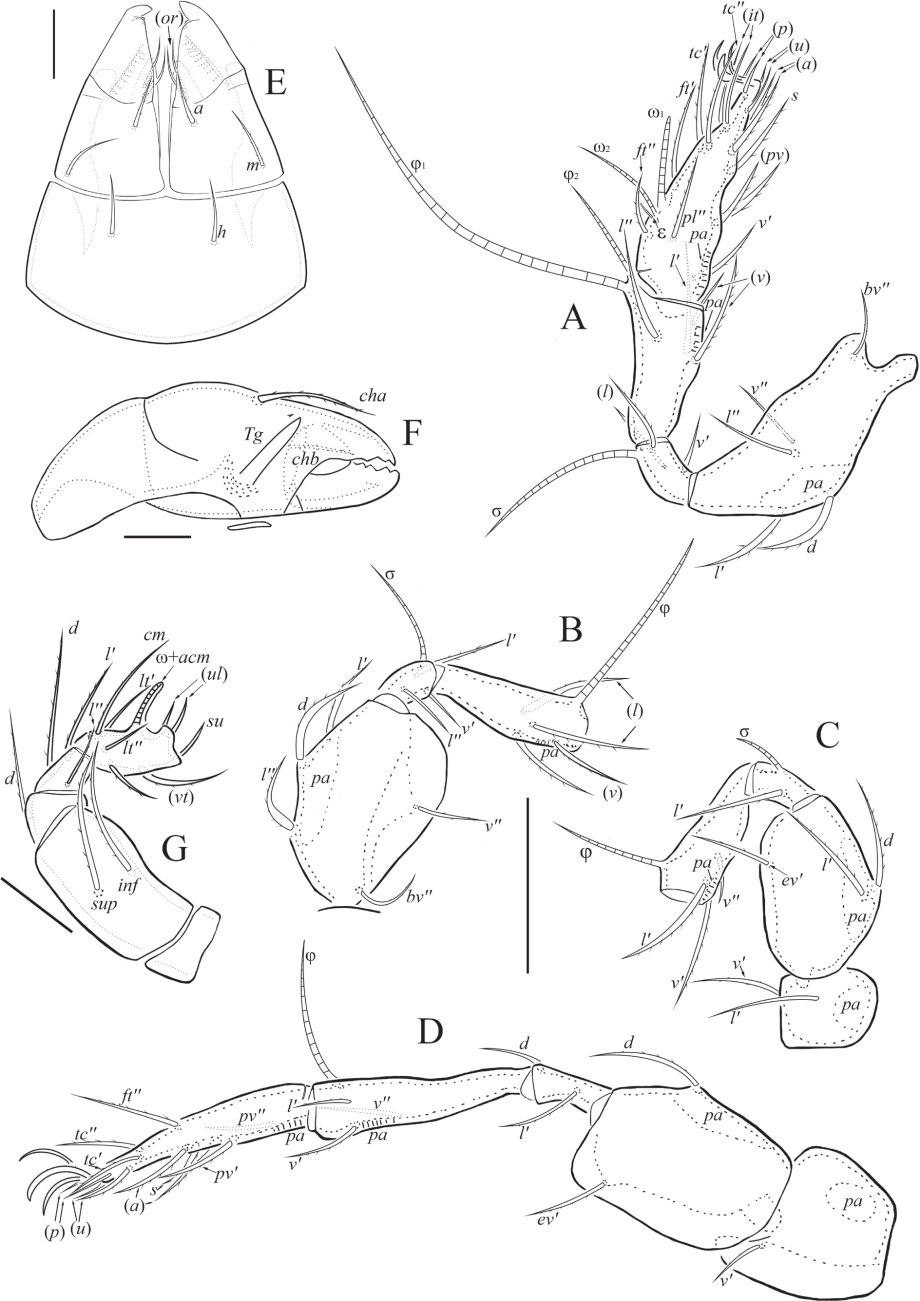
Indoribates (Haplozetes) tajikistanensis sp. nov. **A** leg I (without trochanter), right, antiaxial view **B** leg II (without trochanter and tarsi), right, antiaxial view **C** leg III (without tarsi), right, antiaxial view **D** leg IV, right, antiaxial view **E** subcapitulum, ventral view **F** chelicera, left, paraxial view **G** palp, right, antiaxial view. Scale bars: 50 μm (**A–E**); 20 μm (**E–G**).

Figs 1 , 2 Common name. 塔吉克单翼甲螨

#### Type material.

***Holotype*** • (female CJ-16-036): Tajikistan, Sughd Region, Ghonchi District, Zarnisor Village; 39°55'27"N, 69°08'42"E; 1055 m; moss, grass, and soil; 21 May 2016; Jun Chen leg. ***Paratypes*** • (three females, two males, CJ-16-037): same data as for holotype.

#### Diagnosis.

Body size: 400–471 × 257–298. Rostrum rounded. Rostral, lamellar and interlamellar setae long, setiform, barbed; *le*>*in*>*ro* .Bothridial seta fusiform, with short stalk and slightly longer, barbed head. Tutorium long, with tooth distally. Notogastral setae short, setiform thin, slightly barbed. Four pairs of notogastral saccules with small opening; *Sa* channel elongate, other channels drop-like. All epimeral setae setiform, barbed. Genital and aggenital setae slightly barbed. Both anal setae and adanal setae *ad*_1-2_ roughened. Adanal lyrifissure immediately to anterolateral margin of anal opening.

#### Description.

***Measurements*.** Body length 450 (holotype, female), 430–471 (five paratypes: two males, three females); body width 277 (holotype), 257–298 (five paratypes).

***Integument*.** Body brown. Surface densely microfoveolate (visible only under high magnification in dissected specimens; × 1000); podosomal region and lateral part of prodorsum partially with dense microgranulate cerotegument.

***Prodorsum*** (Fig. 1A, C). Rostrum rounded. Tutorium long, with tooth distally. Lamellae long, located dorsolaterally. Sublamella short. Sublamellar porose area (7) oval. Rostral (57–67), lamellar (73–80) and interlamellar (65–75) setae setiform, barbed. Bothridial seta (49–55) fusiform, with short stalk and slightly longer, barbed head. Exobothridial seta (9) setiform short, roughened. Sejugal porose area diffuse, located posterolateral to interlamellar seta, transversely elongate.

***Notogaster*** (Fig. 1A, C). Anterior notogastral margin convex medially. Dorsophragma elongate. Pteromorph with distinct hinge. Ten pairs of notogastral setae (15–33) setiform, slightly barbed (visible under high magnification). Four pairs of saccules with small opening, *Sa* channel elongate, others channels drop-like. Opisthonotal gland opening and all lyrifissures clearly distinct.

***Gnathosoma*** (Fig. 2E–G). Subcapitulum size: 88–93 × 69–73. Subcapitular setae setiform; *m* (15–17) longer than *a* (13–15) and *h* (12–13); *a* and *m* barbed, *h* smooth. Both adoral setae (7–10) setiform, roughened. Palp (length 59–65) with setation 0-2-1-3-9(+ω). Postpalpal seta (5) spiniform, roughened. Chelicera (length 105–112) with two setiform, barbed setae (*cha*: 34–36; *chb*: 21–23).

***Epimeral and lateral podosomal regions*** (Fig. 1B, C). Epimeral setal formula: 3-1-3-3. All epimeral setae (11–19) setiform, barbed. Pedotectum II trapezoid, rounded apically. Discidium triangular, rounded apically. Circumpedal carina long, reaching to acetabulum II.

***Anogenital region*** (Fig. 1B, C). Genital (*g*_1_ 23, *g*_2_-*g*_5_ 17–11), aggenital (14–16) setae and adanal seta *ad*_3_ setiform, slightly barbed. Both anal setae (9–11) and adanal setae *ad*_1_, *ad*_2_ (11–13) setiform, roughened. Adanal lyrifissure close and anterolateral to anal plate.

***Legs*** (Fig. 2A–D). All legs tridactylous; median claw thick, lateral claws thin; all claws slightly barbed on dorsal side. Dorsoparaxial porose area on femora I–IV and on trochanters III, IV well visible. Formulas of leg setation and solenidia: I (1-5-3-4-18) [1-2-2], II (1-5-3-4-15) [1-1-2], III (2-3-1-3-15) [1-1-0], IV (1-2-2-3-12) [0-1-0]. Homology of setae and solenidia indicated in Table 1.

**Table 1.** Leg setation and solenidia of adult Indoribates (Haplozetes) tajikistanensis sp. nov.

leg	Tr	Fe	Ge	Ti	Ta
I	*v*′	*d*, (*l*), *bv*″, *v*″	(*l*), *v*′, σ	(*l*), (*v*), φ_1_, φ_2_	(*ft*), (*tc*), (*it*), (*p*), (*u*), (*a*), *s*, (*pv*), *pl*″, *v*′, ε, ω_1_, ω_2_
II	*v*′	*d*, (*l*), *bv*″, *v*″	(*l*), *v*′, σ	(*l*), (*v*), φ	(*ft*), (*tc*), (*it*), (*p*), (*u*), (*a*), *s*, (*pv*), ω_1_, ω_2_
III	*l*′, *v*′	*d*, *l*′, *ev*′	*l*′, σ	*l*′, (*v*), φ	(*ft*), (*tc*), (*it*), (*p*), (*u*), (*a*), *s*, (*pv*)
IV	*v*′	*d*, *ev*′	*d*, *l*′	*l*′, (*v*), φ	*ft*″, (*tc*), (*p*), (*u*), (*a*), *s*, (*pv*)

**Note.**Roman letters refer to normal setae, Greek letters to solenidia (except ε = famulus). Single prime (′) marks setae on the anterior and double prime (″) seta on the posterior side of a given leg segment. Parentheses refer to a pair of setae.

#### Etymology.

The specific name *tajikistanensis* refers to the country encompassing the type locality of this new species.

#### Remarks.

Indoribates (Haplozetes) tajikistanensis sp. nov. is morphologically similar to I. (H.) vindobonensis (Willmann, 1935) (see also Grandjean 1936; Beck 1964; Kunst 1977; Mahunka 1982, 2001) from the Palaearctic, Ethiopian, Neotropical, and Oriental regions in having fusiform bothridial setae. However, the new species differs from I. (H.) vindobonensis by the position of the adanal lyrifissure *iad* (anterolateral margin of anal opening vs close and parallel to lateral margin of anal opening) and the shape of the saccule *S2* (with not bilobed channel vs with bilobed channel).

### ﻿ Indoribates (Haplozetes) asetosus sp. nov.

Taxon classificationAnimaliaOribatidaHaplozetidae

7FBACEF7-E316-512E-B669-50C1BDE1BFC5

https://zoobank.org/2033FE1A-4CC5-4C8B-BC4D-C4A46527CE4A

**Figure 3.**
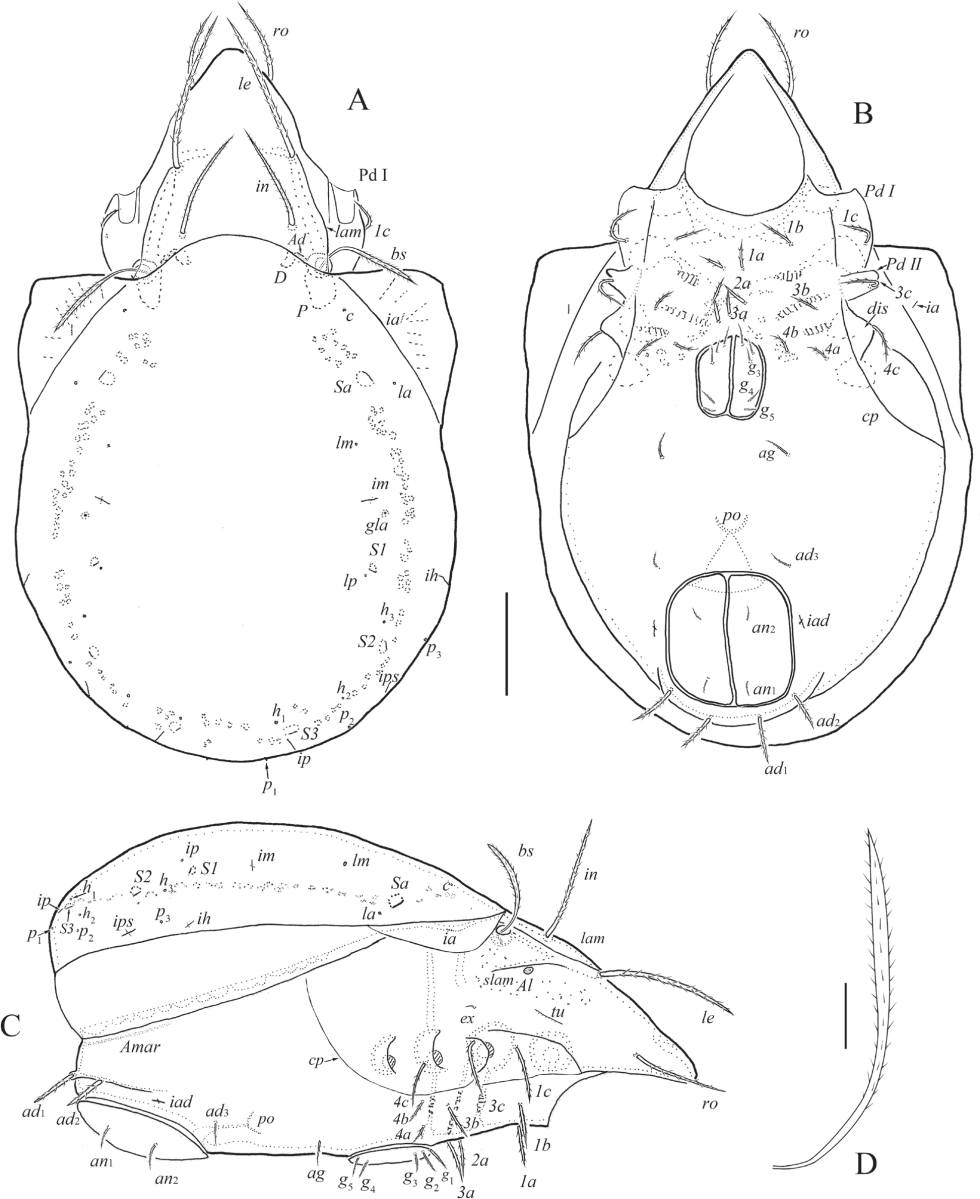
Indoribates (Haplozetes) asetosus sp. nov. **A** dorsal view (legs not shown) **B** ventral view (gnathosoma and legs not shown) **C** lateral view (gnathosoma and legs not shown) **D** bothridial seta. Scale bars: 100 μm (**A–C**); 20 μm (**D**).

**Figure 4.**
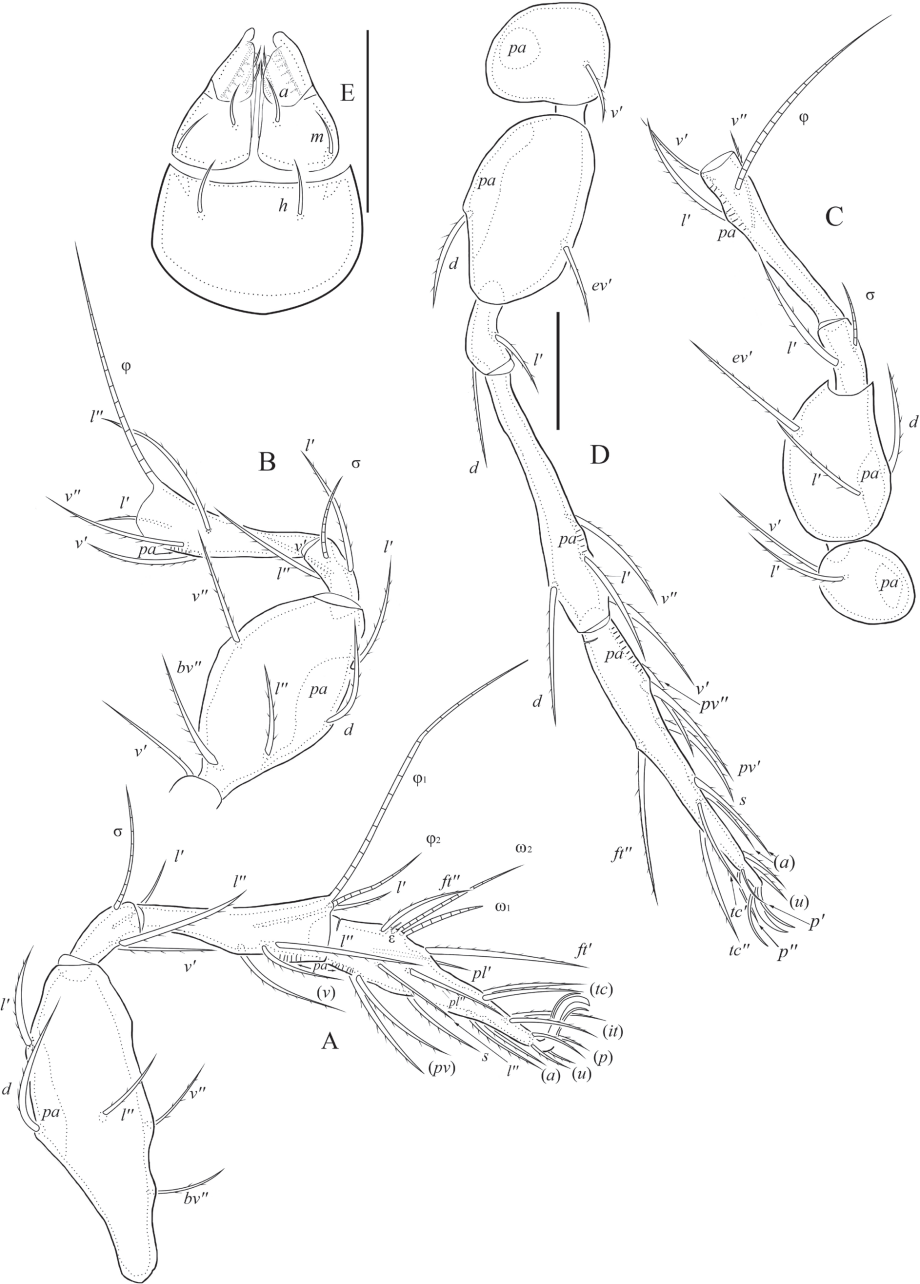
Indoribates (Haplozetes) asetosus sp. nov. **A** leg I (without trochanter), right, antiaxial view **B** leg II (without tarsi), left, antiaxial view **C** leg III (without tarsi), right, antiaxial view **D** leg IV, right, antiaxial view **E** subcapitulum, ventral view. Scale bars: 50 μm (**A–D**); 20 μm (**E**).

Figs 3 , 4 Common name. 无毛单翼甲螨

#### Type material.

***Holotype*** • (female CJ-14-064): Tajikistan, Tavildara District, Hoja Pulod Village, 38°43'28"N, 70°27'14"E, 1730 m, grass and soil, 14 July 2014, Jun Chen leg. ***Paratypes*** • (one female, one male, CJ-14-064): same data as for holotype.

#### Diagnosis.

Body size: 620–740 × 414–480. Rostrum rounded. Rostral, lamellar and interlamellar setae long, setiform, barbed; *le*>*in*>*ro*. Bothridial seta long, with shorter stalk and longer, uniformly thickened, barbed head. Tutorium short, simple. Ten pairs of notogastral setae represented by alveoli. All epimeral setae setiform, slightly thickened, barbed. Genital, aggenital, anal and adanal setae setiform, barbed; adanal setae *ad*_1_ and *ad*_2_ long, thickened, inserted on semicircular protrusion. Solenidion φ on tibia IV absent, but seta *d* developed instead it.

#### Description.

***Measurements*.** Body length 682 (holotype, female), 620–740 (two paratypes: one male, one female); body width 420 (holotype), 414–480 (two paratypes).

***Integument*.** Body light brown. Surface densely microfoveolate (visible only under high magnification in dissected specimens; × 1000); podosomal region and lateral part of prodorsum partially with dense, microgranulate cerotegument.

***Prodorsum*** (Fig. 3A, C). Rostrum rounded. Tutorium short, simple. Lamella about 1/2 length of prodorsum. Sublamella visible. Sublamellar porose area (7) oval. Rostral (86–97), lamellar (138–149) and interlamellar (108–116) setae setiform, barbed. Exobothridial seta (7–9) setiform, thin, smooth. Bothridial seta (100–112) long, with shorter stalk and longer, uniformly thickened, barbed head. Sejugal porose area distinct, located posterolateral to interlamellar seta, transversely elongate.

***Notogaster*** (Fig. 3A, C, D). Anterior notogastral margin convex medially. Dorsophragma nearly oval. Pteromorph with distinct hinge. Ten pairs of notogastral setae represented by alveoli. Four pairs of saccules with small opening and drop-like channel. Opisthonotal gland opening and all lyrifissures distinct.

***Gnathosoma*** (Figs 4E). Subcapitulum size: 151–159 × 105–112. All subcapitular setae (*h* 22–26, *m* 24–26, *a* 24–25) setiform, smooth. Both adoral setae (7–10) setiform, barbed. Palp (length 85–91) with setation 0-2-1-3-9(+ω). Postpalpal seta (7) spiniform, roughened. Chelicera (length 150–162) with two setiform barbed setae (*cha*: 39–44; *chb*: 25–29).

***Epimeral and lateral podosomal regions*** (Fig. 3B, C). Epimeral setal formula: 3-1-3-3. All epimeral setae (30–56) setiform, slightly thickened, barbed. Pedotectum II trapezoid. Discidium triangular, rounded apically. Circumpedal carinae long, reaching to acetabulum II.

***Anogenital region*** (Fig. 3B, C). Genital (15–24), aggenital (21–23) and anal (21–25) setae setiform, barbed. All adanal setae barbed, *ad*_1-2_ (41–45) thicker, inserted on semicircular protrusion; *ad*_3_ (21–23) setiform. Adanal lyrifissure laterally located, adjacent to anal aperture.

***Legs*** (Fig. 4A–D). All legs tridactylous; median claw thick, lateral claws thin; all claws slightly barbed on dorsal side. Dorsoparaxial porose area on femora I–IV and on trochanters III, IV well visible. Formulas of leg setation and solenidia: I (1-5-3-4-19) [1-2-2], II (1-5-3-4-15) [1-1-2], III (2-3-1-3-15) [1-1-0], IV (1-2-2-4-12) [0-0-0]. Homology of setae and solenidia indicated in Table 2. Solenidion φ on tibia IV absent, but seta *d* developed instead it.

**Table 2.** Leg setation and solenidia of adult Indoribates (Haplozetes) asetosus sp. nov.

leg	Tr	Fe	Ge	Ti	Ta
I	*v*′	*d*, (*l*), *bv*″, v″	(*l*), *v*′, σ	(*l*), (*v*), φ_1_, φ_2_	(*ft*), (*tc*), (*it*), (*p*), (*u*), (*a*), *s*, (*pv*), (*pl*), *l*″, ε, ω_1_, ω_2_
II	*v*′	*d*, (*l*), *bv*″, *v*″	(*l*), *v*′, σ	(*l*), (*v*), φ	(*ft*), (*tc*), (*it*), (*p*), (*u*), (*a*), *s*, (*pv*), ω_1_, ω_2_
III	*l*′, *v*′	*d*, *l*′, *ev*′	*l*′, σ	*l*′, (*v*), φ	(*ft*), (*tc*), (*it*), (*p*), (*u*), (*a*), *s*, (*pv*)
IV	*v*′	*d*, *ev*′	*d*, *l*′	*l*′, (*v*), *d*	*ft*″, (*tc*), (*p*), (*u*), (*a*), *s*, (*pv*)

**Note.**See Table 1 for explanation.

#### Etymology.

The specific name *asetosus* refers to notogastral setae represented by alveoli.

#### Remarks.

Indoribates (Haplozetes) asetosus sp. nov. is morphologically similar to I. (H.) fusifer (Berlese, 1908) (see also Akrami and Behmanesh 2013) and I. (H.) triungulatus (Beck, 1964) in having long bothridial seta with narrowly elongate head, but differs from I. (H.) fusifer by the absence of φ on tibia IV (vs presence), and the thickened adanal setae *ad*_1_, *ad*_2_ (vs thin). Moreover, the new species is distinguished from I. (H.) triungulatus by the shape of the interlamellar seta (long, barbed vs short, smooth), the length of the adanal setae *ad*_1-2_ (long vs short) and tutorium (short vs long); and the shape of all notogastral setae (represented by alveoli vs short, setiform).

The most remarkable diagnosis of the new species is that solenidion φ on the leg tibia IV is absent, but represented by *d*. We examined the type specimens carefully and found no solenidion φ and no alveolus; there is a barbed *d* seta in the corresponding position. This feature is not typical for Haplozetidae, but it is typical for representatives of the related oripodoid family Parakalummidae.

## Supplementary Material

XML Treatment for Indoribates (Haplozetes) tajikistanensis

XML Treatment for Indoribates (Haplozetes) asetosus

## ﻿Additional information

### Author ORCIDs

Jun Chen https://orcid.org/0000-0002-0325-2532

### Data availability

All of the data that support the findings of this study are available in the main text.
